# Unsupervised free-breathing 3-dimensional imaging of morphology, function and flow in congenital heart disease under 30 minutes: pilot study

**DOI:** 10.1186/1532-429X-16-S1-W8

**Published:** 2014-01-16

**Authors:** Rajesh Krishnamurthy, Ramkumar Krishnamurthy, Elijah Bolin, LaDonna Malone, Myriam E Almeida-Jones, Amol Pednekar

**Affiliations:** 1 Radiology, Texas Children's Hospital, Houston, Texas, USA; 2 Radiology, Baylor College of Medicine, Houston, Texas, USA; 3 Clinical Science, Philips Healthcare, Houston, Texas, USA

## Background

Cardiac MRI for congenital heart disease (CHD) is an operator dependent and time-intensive examination requiring real-time decision making regarding choice of sequences, planes, and acquisition parameters to adapt to unique morphological and functional variables in a given patient.

## Objective

To evaluate technical feasibility, image quality and quantitative integrity of a free-breathing (FB) protocol following administration of blood pool contrast agent, utilizing 3-dimensional (3D) imaging of morphology, function, and flow without physician supervision in a cohort of patients with CHD.

## Methods

**Figure 1:**
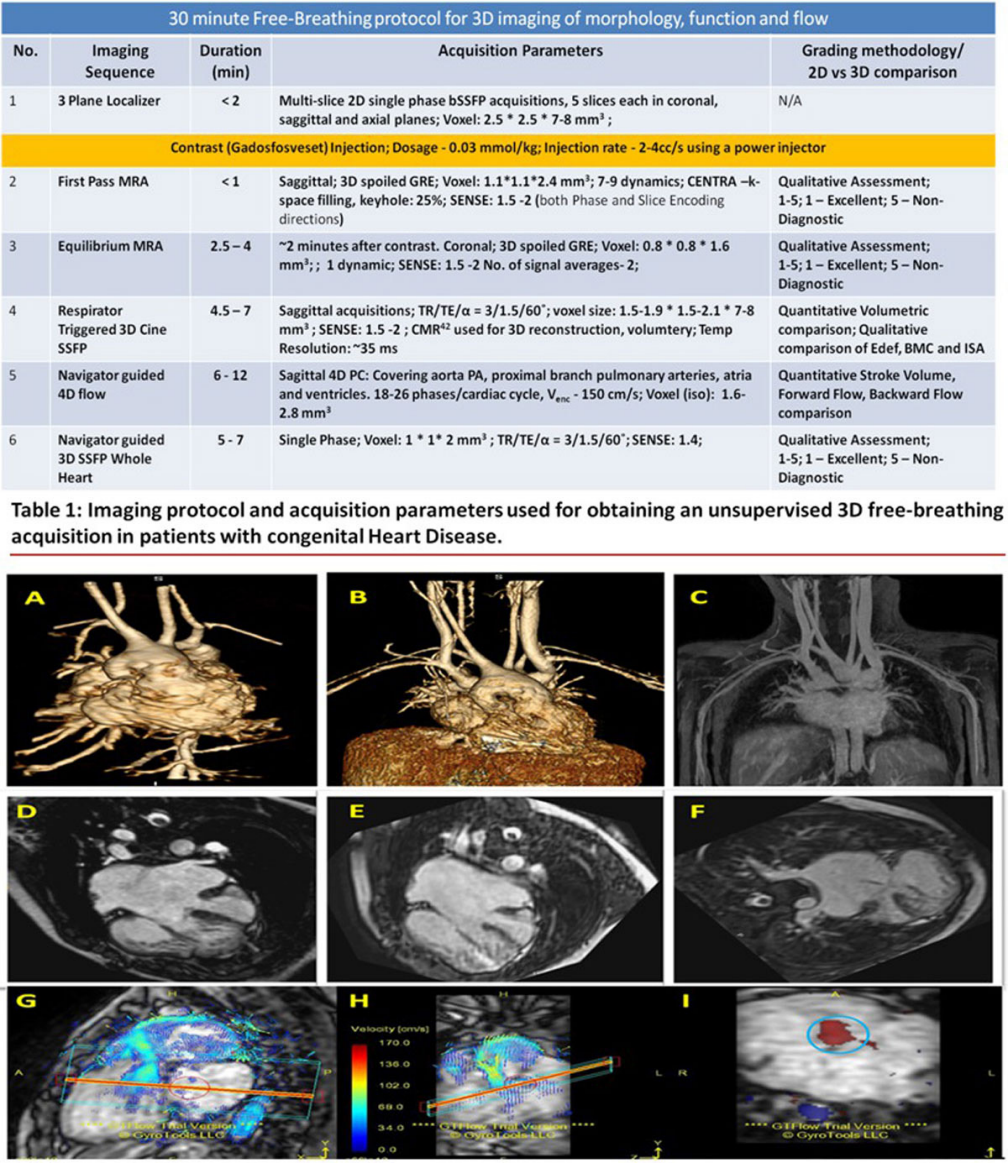
**Representative images of patients acquired using the FB 3D protocol**. First pass MRA 3D MIP acquired immediately after administration of blood pool contrast agent is shown in (A). (B) and (C) are equilibrium MRA images acquired ~2-3 minutes after contrast injection. Additional vasculature is clearly see in equilibrium MRA wrt first pass MRA images. (D) is a 2D SSFP 4-chamber cine image; (E) and (F) are reconstructed 3D images obtained in a similar imaging plan. Regurgitant jet is clearly seen in (F) that could not be clearly visualized using 2D acquisition. (G), (H) and (I) demonstrates feasibility of capturing complex anatomic details/flow (pulmonary stenosis) using 4D flow imaging. Whole heart SSFP imaging (not shown) post contrast also demonstrated significant clinical utility.

**Figure 2:**
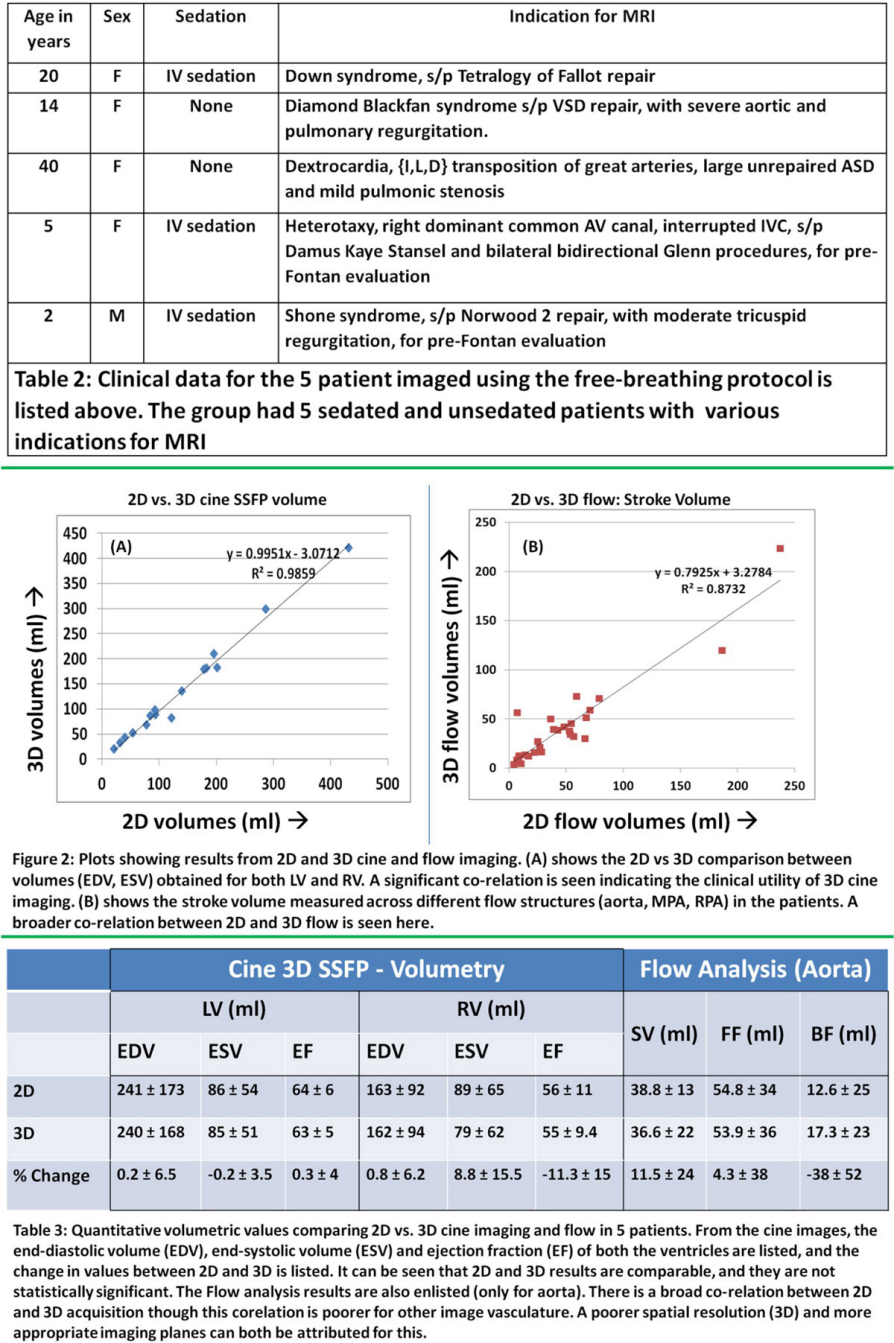


Five patients with CHD were included in this pilot study (table 2 in Figure 2). The FB MR studies were performed on a Philips Acheiva 1.5T magnet using a 5-channel phased array coil (see Table 1 in Figure 1) 1. Respiratory synchronized [1], time-resolved MRA 2. Equilibrium phase MRA 3. 3D cine SSFP 4.4D phase contrast (PC) flow imaging 5.3D whole-heart single phase SSFP (coronary) Comparative data was obtained using conventional 2D cine RT SSFP sequences [2] in the VLA, 4 chamber and short axis planes, and 2D PC imaging. Data Analysis: Image quality assessment and quantitative volumetric and flow analysis were performed by three blinded, experienced users. MRA images were graded using a semi-quantitative scale from 1-5 for relevant imaging targets in CHD [1], with 1: excellent, no limitations, and 5: non-diagnostic. The clinical scoring system for 2D and 3D cine SSFP was based on blood-myocardial contrast, endocardial edge definition and inter-slice alignment [2]. Paired t-test analysis was performed on LV and RV volumes obtained by an experienced observer using the same software

## Results

All FB 3D sequences were technically feasible in all 5 patients. Average time for completion of 5 FB 3D sequences was 29 minutes. Average score for first-pass MRA was 1.9/5. Average score for equilibrium MRA was 1.3/5. Clinical scores for 2D SSFP were consistently better than 3D-SSFP, but 3D SSFP images were adequate for recognition of pathology in all cases (2D vs 3D: 1.5 ± 0.5 vs 1.6 ± 0.9) and had better inter-slice alignment (1.4 ± 0.5 vs 1 ± 0). Average percentage difference between 2D and 3D cine SSFP volumetric data is shown in table 3, and Figure 2. Comparative flow analysis between 2D PC and 4D PC data revealed broad correlation (Figure 2, table 3) though the stroke volume, forward and backward flows through the aorta were not statistically different (p > 0.35; paired Student's t-test)

## Conclusions

The free breathing first pass MRA, equilibrium MRA, 3D cine SSFP, and 3D single-phase SSFP exhibit significant clinical utility. We demonstrate the feasibility of performing an observer independent comprehensive CMR in CHD utilizing FB 3D acquisitions for morphology, function and flow within 30 minutes using a 5-channel phased-array coil. Better acquisition hardware (eg., 32 ch coil) will lead to superior image quality.

## Funding

None.
